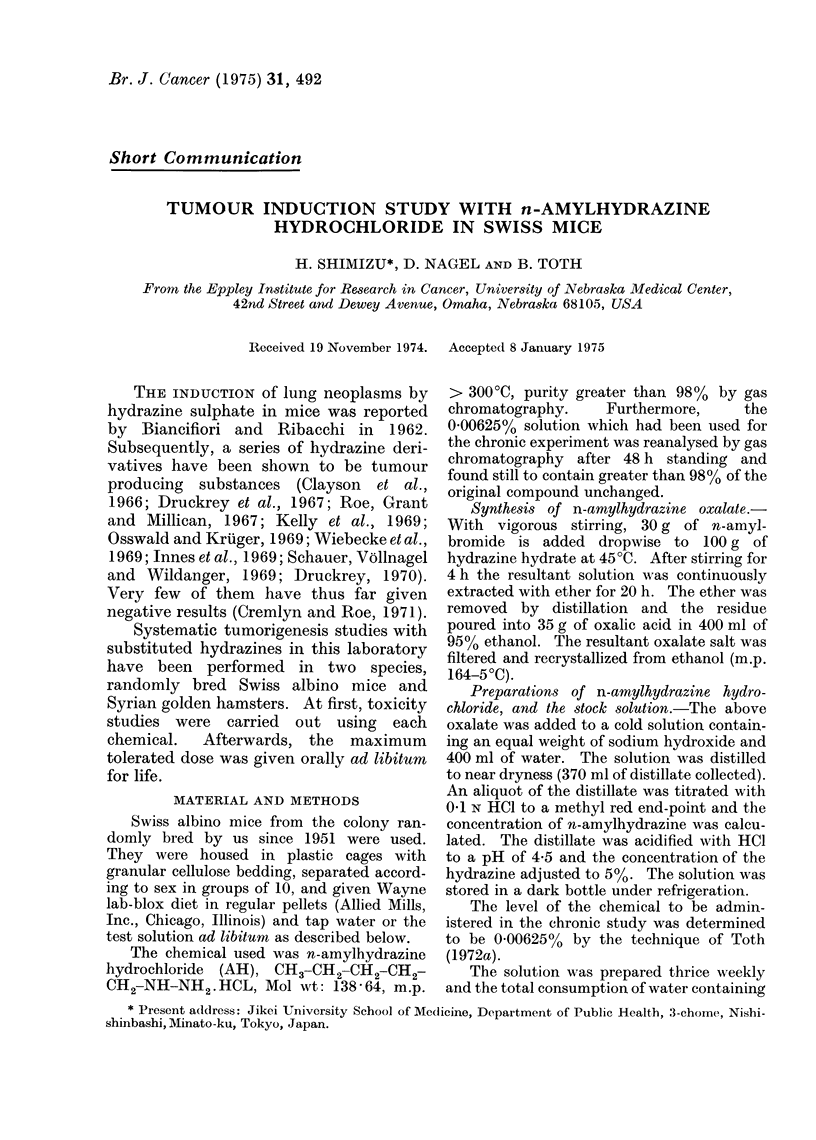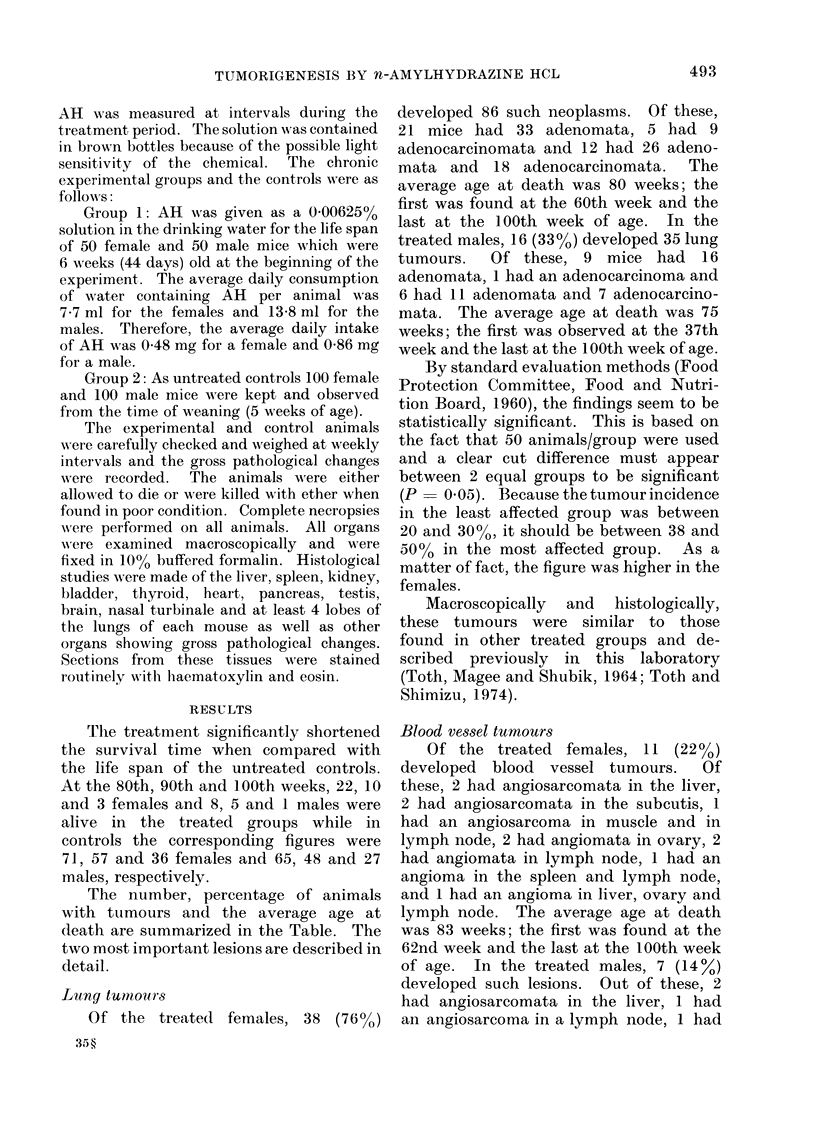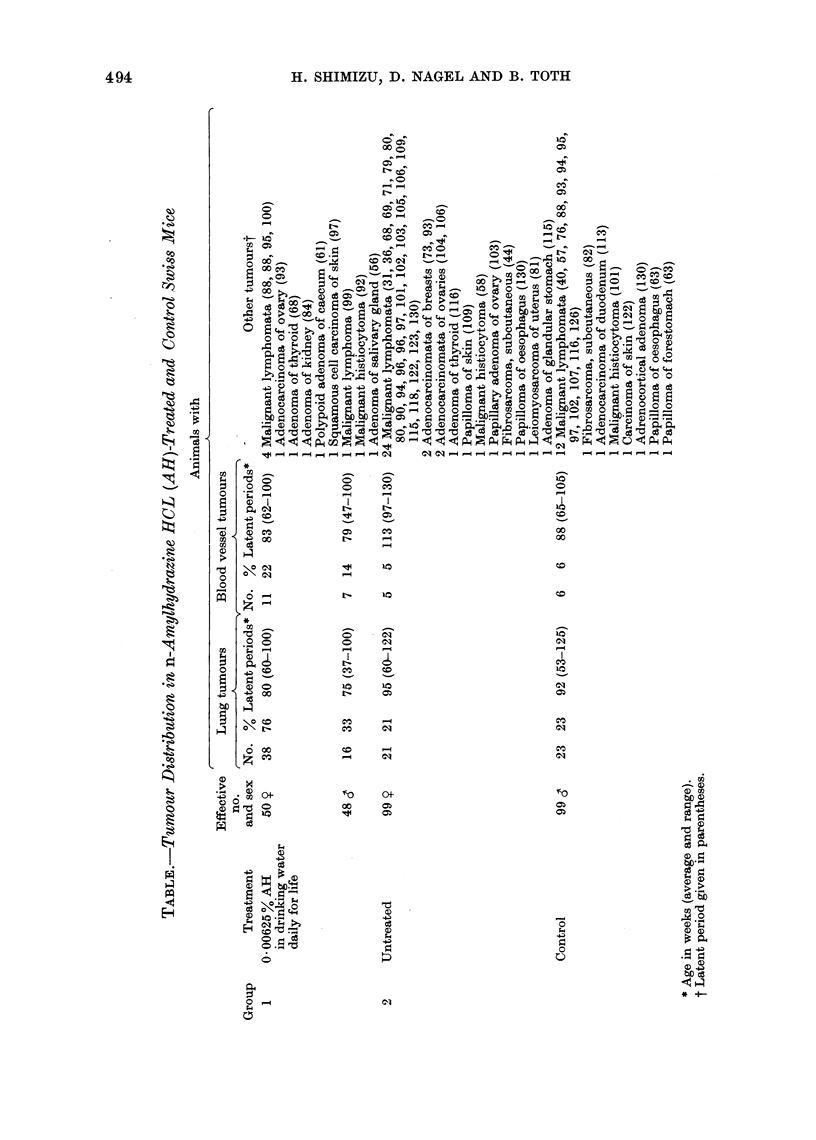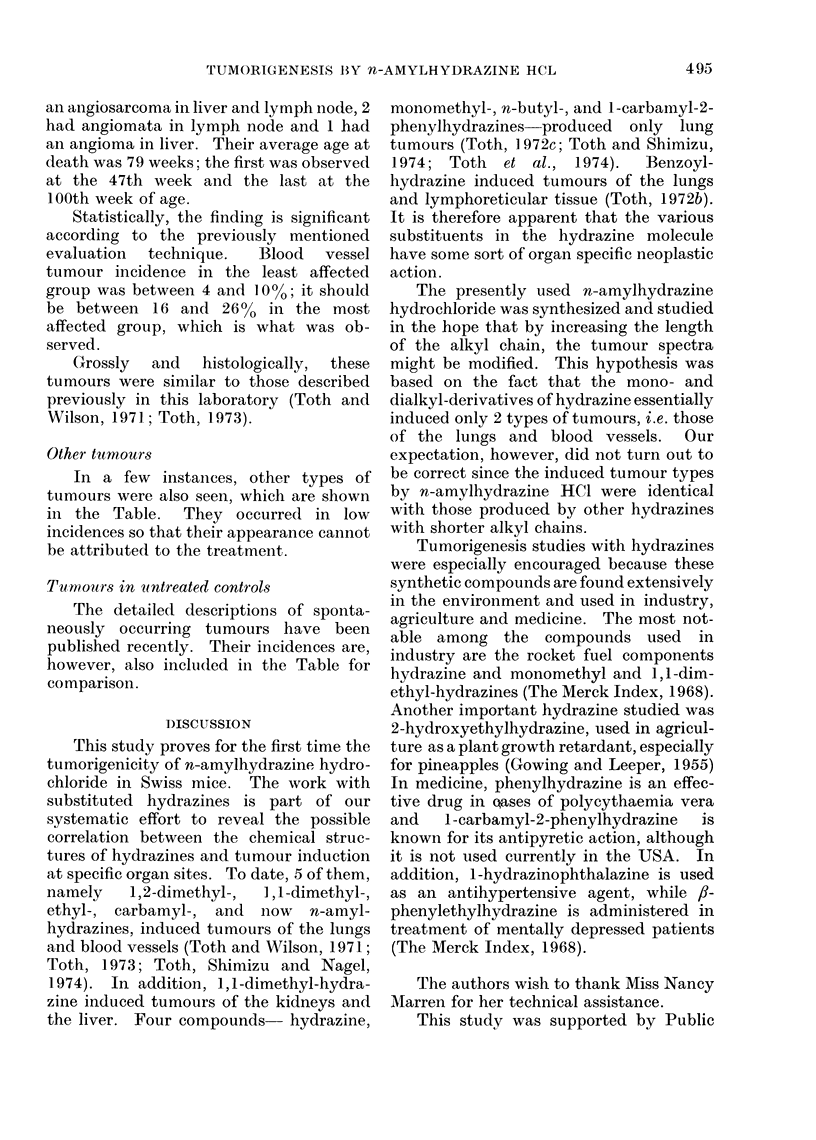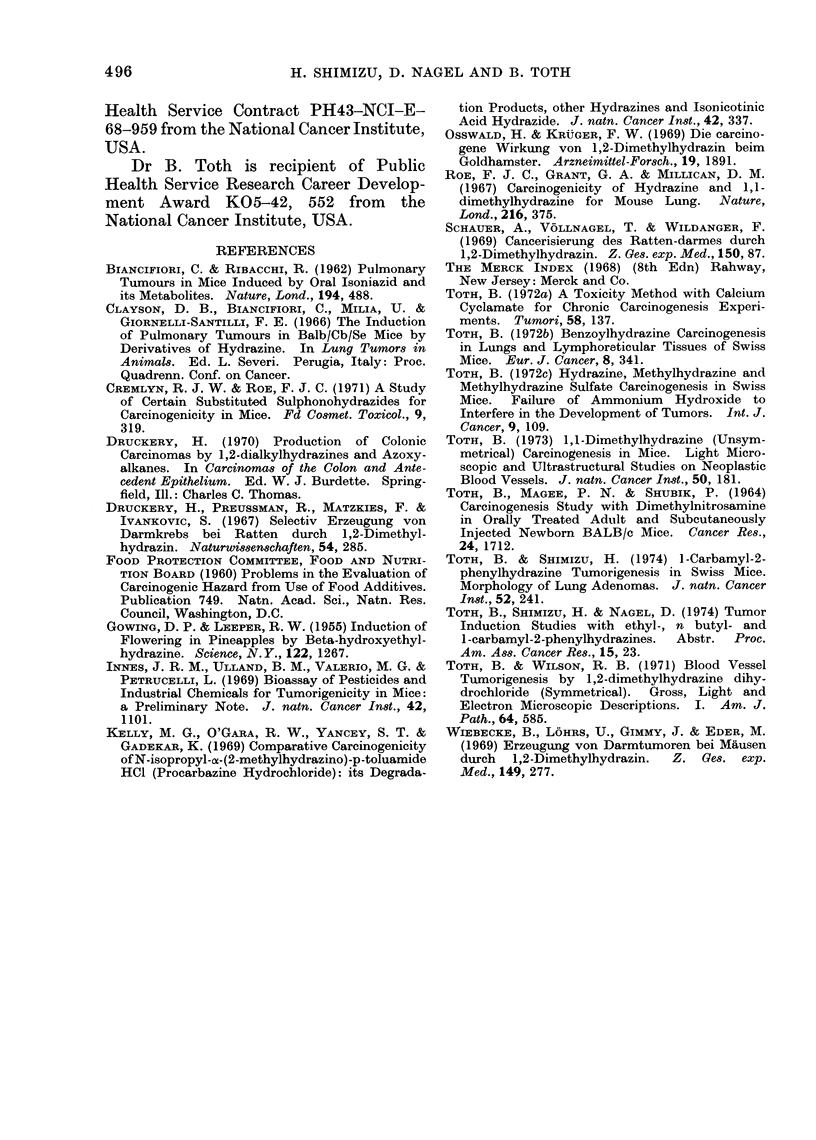# Tumour induction study with n-amylhydrazine hydrochloride in Swiss mice.

**DOI:** 10.1038/bjc.1975.89

**Published:** 1975-04

**Authors:** H. Shimizu, D. Nagel, B. Toth


*Br. J. Cancer* (1975) **31**, 492

***Short Communication***

### TUMOUR INDUCTION STUDY WITH *n*-AMYLHYDRAZINE HYDROCHLORIDE IN SWISS MICE

H. SHIMIZU*, D. NAGEL AND B. TOTH

*From the Eppley Institute for Research in Cancer, University of Nebraska Medical Center, 42nd Street and Dewey Avenue, Omaha, Nebraska* 68105, *USA*

Received 19 November 1974. Accepted 8 January 1975

THE INDUCTION of lung neoplasms by hydrazine sulphate in mice was reported by Biancifiori and Ribacchi in 1962. Subsequently, a series of hydrazine derivatives have been shown to be tumour producing substances (Clayson *et al.*, 1966; Druckrey *et al.*, 1967; Roe, Grant and Millican, 1967; Kelly *et al.*, 1969; Osswald and Krüger, 1969; Wiebecke *et al.*, 1969; Innes *et al.*, 1969; Schauer, Völlnagel and Wildanger, 1969; Druckrey, 1970). Very few of them have thus far given negative results (Cremlyn and Roe, 1971).

Systematic tumorigenesis studies with substituted hydrazines in this laboratory have been performed in two species, randomly bred Swiss albino mice and Syrian golden hamsters. At first, toxicity studies were carried out using each chemical. Afterwards, the maximum tolerated dose was given orally *ad libitum* for life.

### MATERIAL AND METHODS

Swiss albino mice from the colony randomly bred by us since 1951 were used. They were housed in plastic cages with granular cellulose bedding, separated according to sex in groups of 10, and given Wayne lab-blox diet in regular pellets (Allied Mills, Inc., Chicago, Illinois) and tap water or the test solution *ad libitum* as described below.

The chemical used was *n*-amylhydrazine hydrochloride (AH), $CH_3$–$CH_2$–$CH_2$–$CH_2$–$CH_2$–NH–$NH_2$.HCL, Mol wt: 138·64, m.p. > 300°C, purity greater than 98% by gas chromatography. Furthermore, the 0·00625% solution which had been used for the chronic experiment was reanalysed by gas chromatography after 48 h standing and found still to contain greater than 98% of the original compound unchanged.

*Synthesis of* n-*amylhydrazine oxalate.*—With vigorous stirring, 30 g of *n*-amylbromide is added dropwise to 100 g of hydrazine hydrate at 45°C. After stirring for 4 h the resultant solution was continuously extracted with ether for 20 h. The ether was removed by distillation and the residue poured into 35 g of oxalic acid in 400 ml of 95% ethanol. The resultant oxalate salt was filtered and recrystallized from ethanol (m.p. 164–5°C).

*Preparations of* n-*amylhydrazine hydrochloride, and the stock solution.*—The above oxalate was added to a cold solution containing an equal weight of sodium hydroxide and 400 ml of water. The solution was distilled to near dryness (370 ml of distillate collected). An aliquot of the distillate was titrated with 0·1 N HCl to a methyl red end-point and the concentration of *n*-amylhydrazine was calculated. The distillate was acidified with HCl to a pH of 4·5 and the concentration of the hydrazine adjusted to 5%. The solution was stored in a dark bottle under refrigeration.

The level of the chemical to be administered in the chronic study was determined to be 0·00625% by the technique of Toth (1972*a*).

The solution was prepared thrice weekly and the total consumption of water containing

* Present address: Jikei University School of Medicine, Department of Public Health, 3-chome, Nishi-shinbashi, Minato-ku, Tokyo, Japan.

AH was measured at intervals during the treatment period. The solution was contained in brown bottles because of the possible light sensitivity of the chemical. The chronic experimental groups and the controls were as follows:

Group 1: AH was given as a 0·00625% solution in the drinking water for the life span of 50 female and 50 male mice which were 6 weeks (44 days) old at the beginning of the experiment. The average daily consumption of water containing AH per animal was 7·7 ml for the females and 13·8 ml for the males. Therefore, the average daily intake of AH was 0·48 mg for a female and 0·86 mg for a male.

Group 2: As untreated controls 100 female and 100 male mice were kept and observed from the time of weaning (5 weeks of age).

The experimental and control animals were carefully checked and weighed at weekly intervals and the gross pathological changes were recorded. The animals were either allowed to die or were killed with ether when found in poor condition. Complete necropsies were performed on all animals. All organs were examined macroscopically and were fixed in 10% buffered formalin. Histological studies were made of the liver, spleen, kidney, bladder, thyroid, heart, pancreas, testis, brain, nasal turbinale and at least 4 lobes of the lungs of each mouse as well as other organs showing gross pathological changes. Sections from these tissues were stained routinely with haematoxylin and eosin.

### RESULTS

The treatment significantly shortened the survival time when compared with the life span of the untreated controls. At the 80th, 90th and 100th weeks, 22, 10 and 3 females and 8, 5 and 1 males were alive in the treated groups while in controls the corresponding figures were 71, 57 and 36 females and 65, 48 and 27 males, respectively.

The number, percentage of animals with tumours and the average age at death are summarized in the Table. The two most important lesions are described in detail.

### *Lung tumours*

Of the treated females, 38 (76%) developed 86 such neoplasms. Of these, 21 mice had 33 adenomata, 5 had 9 adenocarcinomata and 12 had 26 adenomata and 18 adenocarcinomata. The average age at death was 80 weeks; the first was found at the 60th week and the last at the 100th week of age. In the treated males, 16 (33%) developed 35 lung tumours. Of these, 9 mice had 16 adenomata, 1 had an adenocarcinoma and 6 had 11 adenomata and 7 adenocarcinomata. The average age at death was 75 weeks; the first was observed at the 37th week and the last at the 100th week of age.

By standard evaluation methods (Food Protection Committee, Food and Nutrition Board, 1960), the findings seem to be statistically significant. This is based on the fact that 50 animals/group were used and a clear cut difference must appear between 2 equal groups to be significant ($P = 0·05$). Because the tumour incidence in the least affected group was between 20 and 30%, it should be between 38 and 50% in the most affected group. As a matter of fact, the figure was higher in the females.

Macroscopically and histologically, these tumours were similar to those found in other treated groups and described previously in this laboratory (Toth, Magee and Shubik, 1964; Toth and Shimizu, 1974).

### *Blood vessel tumours*

Of the treated females, 11 (22%) developed blood vessel tumours. Of these, 2 had angiosarcomata in the liver, 2 had angiosarcomata in the subcutis, 1 had an angiosarcoma in muscle and in lymph node, 2 had angiomata in ovary, 2 had angiomata in lymph node, 1 had an angioma in the spleen and lymph node, and 1 had an angioma in liver, ovary and lymph node. The average age at death was 83 weeks; the first was found at the 62nd week and the last at the 100th week of age. In the treated males, 7 (14%) developed such lesions. Out of these, 2 had angiosarcomata in the liver, 1 had an angiosarcoma in a lymph node, 1 had

TABLE.—*Tumour Distribution in* n-*Amylhydrazine HCL (AH)-Treated and Control Swiss Mice*

| | | | Animals with | | | | | | |
|---|---|---|---|---|---|---|---|---|---|
| | | Effective no. | Lung tumours | | | Blood vessel tumours | | | |
| Group | Treatment | and sex | No. | % | Latent periods* | No. | % | Latent periods* | Other tumours† |
| 1 | 0·00625% AH in drinking water daily for life | 50 ♀ | 38 | 76 | 80 (60–100) | 11 | 22 | 83 (62–100) | 4 Malignant lymphomata (88, 88, 95, 100)<br>1 Adenocarcinoma of ovary (93)<br>1 Adenoma of thyroid (68)<br>1 Adenoma of kidney (84)<br>1 Polypoid adenoma of caecum (61)<br>1 Squamous cell carcinoma of skin (97) |
| | | 48 ♂ | 16 | 33 | 75 (37–100) | 7 | 14 | 79 (47–100) | 1 Malignant lymphoma (99)<br>1 Malignant histiocytoma (92)<br>1 Adenoma of salivary gland (56) |
| 2 | Untreated | 99 ♀ | 21 | 21 | 95 (60–122) | 5 | 5 | 113 (97–130) | 24 Malignant lymphomata (31, 36, 68, 69, 71, 79, 80, 80, 90, 94, 96, 96, 97, 101, 102, 103, 105, 106, 109, 115, 118, 122, 123, 130)<br>2 Adenocarcinomata of breasts (73, 93)<br>2 Adenocarcinomata of ovaries (104, 106)<br>1 Adenoma of thyroid (116)<br>1 Papilloma of skin (109)<br>1 Malignant histiocytoma (58)<br>1 Papillary adenoma of ovary (103)<br>1 Fibrosarcoma, subcutaneous (44)<br>1 Papilloma of oesophagus (130)<br>1 Leiomyosarcoma of uterus (81)<br>1 Adenoma of glandular stomach (115) |
| | Control | 99 ♂ | 23 | 23 | 92 (53–125) | 6 | 6 | 88 (65–105) | 12 Malignant lymphomata (40, 57, 76, 88, 93, 94, 95, 97, 102, 107, 116, 126)<br>1 Fibrosarcoma, subcutaneous (82)<br>1 Adenocarcinoma of duodenum (113)<br>1 Malignant histiocytoma (101)<br>1 Carcinoma of skin (122)<br>1 Adrenocortical adenoma (130)<br>1 Papilloma of oesophagus (63)<br>1 Papilloma of forestomach (63) |

* Age in weeks (average and range).
† Latent period given in parentheses.

an angiosarcoma in liver and lymph node, 2 had angiomata in lymph node and 1 had an angioma in liver. Their average age at death was 79 weeks; the first was observed at the 47th week and the last at the 100th week of age.

Statistically, the finding is significant according to the previously mentioned evaluation technique. Blood vessel tumour incidence in the least affected group was between 4 and 10%; it should be between 16 and 26% in the most affected group, which is what was observed.

Grossly and histologically, these tumours were similar to those described previously in this laboratory (Toth and Wilson, 1971; Toth, 1973).

*Other tumours*

In a few instances, other types of tumours were also seen, which are shown in the Table. They occurred in low incidences so that their appearance cannot be attributed to the treatment.

*Tumours in untreated controls*

The detailed descriptions of spontaneously occurring tumours have been published recently. Their incidences are, however, also included in the Table for comparison.

### DISCUSSION

This study proves for the first time the tumorigenicity of $n$-amylhydrazine hydrochloride in Swiss mice. The work with substituted hydrazines is part of our systematic effort to reveal the possible correlation between the chemical structures of hydrazines and tumour induction at specific organ sites. To date, 5 of them, namely 1,2-dimethyl-, 1,1-dimethyl-, ethyl-, carbamyl-, and now $n$-amylhydrazines, induced tumours of the lungs and blood vessels (Toth and Wilson, 1971; Toth, 1973; Toth, Shimizu and Nagel, 1974). In addition, 1,1-dimethyl-hydrazine induced tumours of the kidneys and the liver. Four compounds— hydrazine, monomethyl-, $n$-butyl-, and 1-carbamyl-2-phenylhydrazines—produced only lung tumours (Toth, 1972*c*; Toth and Shimizu, 1974; Toth *et al.*, 1974). Benzoylhydrazine induced tumours of the lungs and lymphoreticular tissue (Toth, 1972*b*). It is therefore apparent that the various substituents in the hydrazine molecule have some sort of organ specific neoplastic action.

The presently used $n$-amylhydrazine hydrochloride was synthesized and studied in the hope that by increasing the length of the alkyl chain, the tumour spectra might be modified. This hypothesis was based on the fact that the mono- and dialkyl-derivatives of hydrazine essentially induced only 2 types of tumours, *i.e.* those of the lungs and blood vessels. Our expectation, however, did not turn out to be correct since the induced tumour types by $n$-amylhydrazine HCl were identical with those produced by other hydrazines with shorter alkyl chains.

Tumorigenesis studies with hydrazines were especially encouraged because these synthetic compounds are found extensively in the environment and used in industry, agriculture and medicine. The most notable among the compounds used in industry are the rocket fuel components hydrazine and monomethyl and 1,1-dimethyl-hydrazines (The Merck Index, 1968). Another important hydrazine studied was 2-hydroxyethylhydrazine, used in agriculture as a plant growth retardant, especially for pineapples (Gowing and Leeper, 1955) In medicine, phenylhydrazine is an effective drug in cases of polycythaemia vera and 1-carbamyl-2-phenylhydrazine is known for its antipyretic action, although it is not used currently in the USA. In addition, 1-hydrazinophthalazine is used as an antihypertensive agent, while $\beta$-phenylethylhydrazine is administered in treatment of mentally depressed patients (The Merck Index, 1968).

The authors wish to thank Miss Nancy Marren for her technical assistance.

This study was supported by Public

Health Service Contract PH43–NCI–E–68–959 from the National Cancer Institute, USA.

Dr B. Toth is recipient of Public Health Service Research Career Development Award KO5–42, 552 from the National Cancer Institute, USA.

### REFERENCES

BIANCIFIORI, C. & RIBACCHI, R. (1962) Pulmonary Tumours in Mice Induced by Oral Isoniazid and its Metabolites. *Nature, Lond.*, **194**, 488.

CLAYSON, D. B., BIANCIFIORI, C., MILIA, U. & GIORNELLI-SANTILLI, F. E. (1966) The Induction of Pulmonary Tumours in Balb/Cb/Se Mice by Derivatives of Hydrazine. In *Lung Tumors in Animals.* Ed. L. Severi. Perugia, Italy: Proc. Quadrenn. Conf. on Cancer.

CREMLYN, R. J. W. & ROE, F. J. C. (1971) A Study of Certain Substituted Sulphonohydrazides for Carcinogenicity in Mice. *Fd Cosmet. Toxicol.*, **9**, 319.

DRUCKERY, H. (1970) Production of Colonic Carcinomas by 1,2-dialkylhydrazines and Azoxyalkanes. In *Carcinomas of the Colon and Antecedent Epithelium.* Ed. W. J. Burdette. Springfield, Ill.: Charles C. Thomas.

DRUCKERY, H., PREUSSMAN, R., MATZKIES, F. & IVANKOVIC, S. (1967) Selectiv Erzeugung von Darmkrebs bei Ratten durch 1,2-Dimethylhydrazin. *Naturwissenschaften*, **54**, 285.

FOOD PROTECTION COMMITTEE, FOOD AND NUTRITION BOARD (1960) Problems in the Evaluation of Carcinogenic Hazard from Use of Food Additives. Publication 749. Natn. Acad. Sci., Natn. Res. Council, Washington, D.C.

GOWING, D. P. & LEEPER, R. W. (1955) Induction of Flowering in Pineapples by Beta-hydroxyethylhydrazine. *Science, N.Y.*, **122**, 1267.

INNES, J. R. M., ULLAND, B. M., VALERIO, M. G. & PETRUCELLI, L. (1969) Bioassay of Pesticides and Industrial Chemicals for Tumorigenicity in Mice: a Preliminary Note. *J. natn. Cancer Inst.*, **42**, 1101.

KELLY, M. G., O'GARA, R. W., YANCEY, S. T. & GADEKAR, K. (1969) Comparative Carcinogenicity of N-isopropyl-α-(2-methylhydrazino)-p-toluamide HCl (Procarbazine Hydrochloride): its Degradation Products, other Hydrazines and Isonicotinic Acid Hydrazide. *J. natn. Cancer Inst.*, **42**, 337.

OSSWALD, H. & KRÜGER, F. W. (1969) Die carcinogene Wirkung von 1,2-Dimethylhydrazin beim Goldhamster. *Arzneimittel-Forsch.*, **19**, 1891.

ROE, F. J. C., GRANT, G. A. & MILLICAN, D. M. (1967) Carcinogenicity of Hydrazine and 1,1-dimethylhydrazine for Mouse Lung. *Nature, Lond.*, **216**, 375.

SCHAUER, A., VÖLLNAGEL, T. & WILDANGER, F. (1969) Cancerisierung des Ratten-darmes durch 1,2-Dimethylhydrazin. *Z. Ges. exp. Med.*, **150**, 87.

THE MERCK INDEX (1968) (8th Edn) Rahway, New Jersey: Merck and Co.

TOTH, B. (1972*a*) A Toxicity Method with Calcium Cyclamate for Chronic Carcinogenesis Experiments. *Tumori*, **58**, 137.

TOTH, B. (1972*b*) Benzoylhydrazine Carcinogenesis in Lungs and Lymphoreticular Tissues of Swiss Mice. *Eur. J. Cancer*, **8**, 341.

TOTH, B. (1972*c*) Hydrazine, Methylhydrazine and Methylhydrazine Sulfate Carcinogenesis in Swiss Mice. Failure of Ammonium Hydroxide to Interfere in the Development of Tumors. *Int. J. Cancer*, **9**, 109.

TOTH, B. (1973) 1,1-Dimethylhydrazine (Unsymmetrical) Carcinogenesis in Mice. Light Microscopic and Ultrastructural Studies on Neoplastic Blood Vessels. *J. natn. Cancer Inst.*, **50**, 181.

TOTH, B., MAGEE, P. N. & SHUBIK, P. (1964) Carcinogenesis Study with Dimethylnitrosamine in Orally Treated Adult and Subcutaneously Injected Newborn BALB/c Mice. *Cancer Res.*, **24**, 1712.

TOTH, B. & SHIMIZU, H. (1974) 1-Carbamyl-2-phenylhydrazine Tumorigenesis in Swiss Mice. Morphology of Lung Adenomas. *J. natn. Cancer Inst.*, **52**, 241.

TOTH, B., SHIMIZU, H. & NAGEL, D. (1974) Tumor Induction Studies with ethyl-, *n* butyl- and 1-carbamyl-2-phenylhydrazines. Abstr. *Proc. Am. Ass. Cancer Res.*, **15**, 23.

TOTH, B. & WILSON, R. B. (1971) Blood Vessel Tumorigenesis by 1,2-dimethylhydrazine dihydrochloride (Symmetrical). Gross, Light and Electron Microscopic Descriptions. I. *Am. J. Path.*, **64**, 585.

WIEBECKE, B., LÖHRS, U., GIMMY, J. & EDER, M. (1969) Erzeugung von Darmtumoren bei Mäusen durch 1,2-Dimethylhydrazin. *Z. Ges. exp. Med.*, **149**, 277.